# In memory of Prof. C. C. Li

**DOI:** 10.1007/s13238-018-0583-y

**Published:** 2018-10-23

**Authors:** Zhi Xia, Juan Tian, Xiaoling Wang, Huanming Yang

**Affiliations:** BGI-Shenzhen, Shenzhen, 518083 China

Professor Ching Chun Li (C. C. Li), one of the greatest geneticists in the world and a pioneer of genetics in China, passed away fifteen years ago (Fig. 1). Let us remember him together.

**Figure 1:**
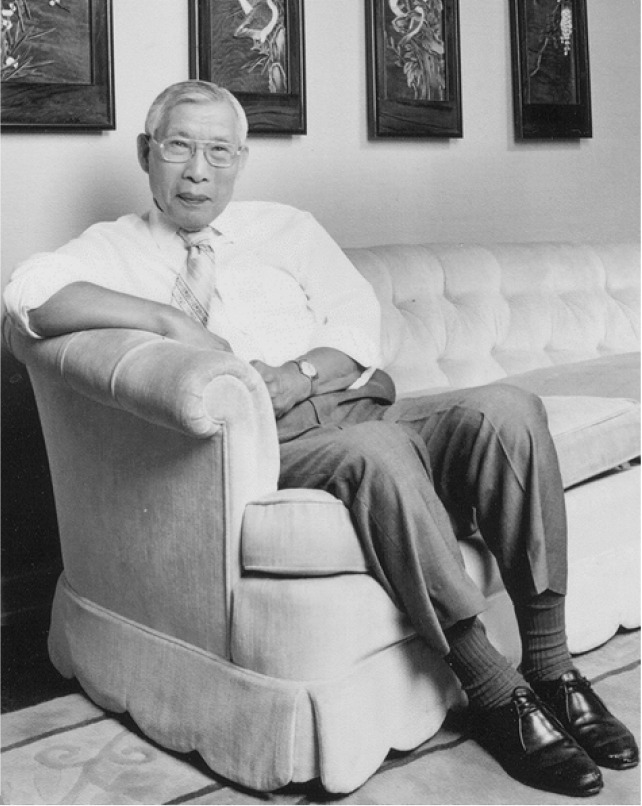
**Ching Chun Li (1912–2003)**

“Do you know who the greatest, world-renown Chinese geneticist is?”

The answer, “C. C. Li, of course!” would puzzle most, if not all, of us; many in our generation don’t know Who is Who (Attention! Li, not Lee, the latter of which was generally used as the general spelling of that generation) (Fig. 2).

**Figure 2:**
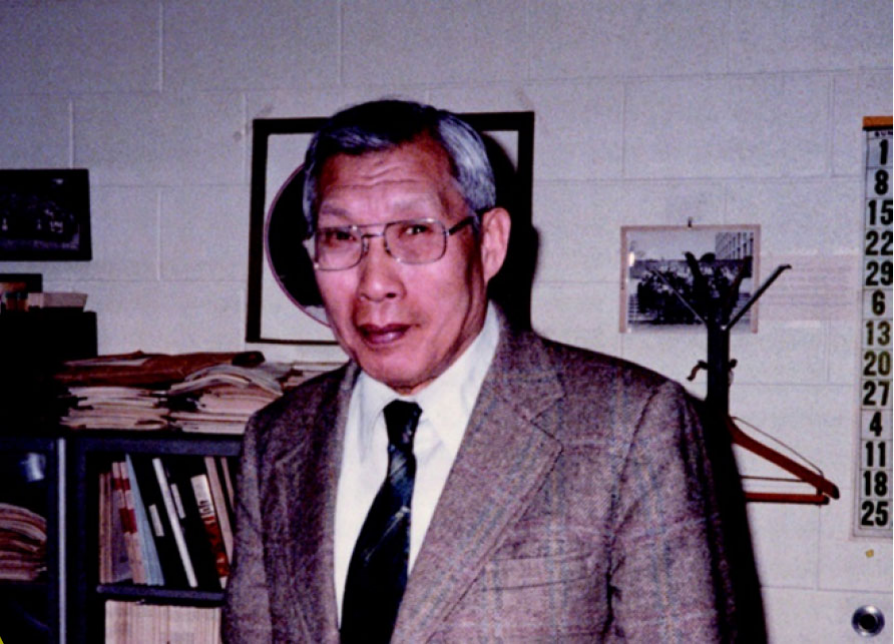
**C. C. Li on the occasion of his seventieth birthday in 1982**

The reason for this answer is simple: Either the individual does not know the history of genetics in or outside mainland China. To be fair, many of us really don’t know so much about the history of genetics in those exceptional or specific historic periods in China.

C. C. Li’s life was full of ups and downs. It has been reported by those who have known him and those who have known of him. This article, with the sole purpose of memorializing him, focuses on three choices which have made the most dramatic changes of his life in 1941, 1949 and 1951, respectively, based on a detailed Chronicle Vitae we try to make with reference to all the materials we are able to collect as Supplement to this article (Petechuk, 1989; Yang, 2004).

(1) 1941—The first choice: To return to his motherland.

In September 1941, Mr. C. C. Li, together with his bride, Clara Lem, took a Dutch ocean liner from San Diego to Hong Kong in order to reach China (Guo, 2016); the purpose of this trip was either for a honeymoon and family visit, or to start his academic career in his motherland (ASHG, on the cover, 2012).

It was the first choice C. C. Li made which changed his life.

All those who have known C. C. Li would have had the same impression: He was curious about everything. Ever since he learned how to read, he read about everything in the world, especially everything related to China. It would be wrong to conclude that he did not know the War against Japanese Invasion was at a climax.

Immediately, he and his wife, together with all the passengers on the liner, experienced what that war brought to the people: The ocean liner, which was supposed to sail for 15 days, was left drifting on ocean for 51 days—even by changing ships—before arriving in Hong Kong on December 6, 1941, just one day before the “Sudden Attack on Pearl Harbor” took place (Guo, 2016).

At that time, it was impossible for C. C. Li to get back to the US, mainly because his wife was pregnant. Her own family had business in the US and expected them to go back (Spiess, 1983, 2005). More horribly, he and his wife were still in the shadow of being “nearly starved to death” from their time on the ocean liner and in Hong Kong. No more starvation!

Unsurprisingly, the couple faced many difficulties on the way to the “Bases for Free China”, or unoccupied part of China. It turned out, from the very beginning, to be a long journey which could still be impossible for us to imagine! However, C. C. Li was determined. He continued, walking and walking, with an empty stomach most of the time; his pregnant wife was carried on a sedan chair almost all the time, which emptied his small wallet. It took them 38 days to travel from Kowlong to Guilin on foot (Chen & Tai, 1998; Spiess, 1983, 2005).

The following quotations from him might help us understand how difficult it was and how determined he was:

“During this time, I learned about hunger… when you are starving, you really can’t do anything. You can’t think of anything but food. You just lie there like zombie. When you see a thing, the first question in your mind is: Is this edible? If it is not, it is useless… I thought all stores should sell food, that’s what starvation does to your thinking… Everything else is trivial and boring. Even death is trivial.” He described these thoughts after arriving in Huiyang (Phi Lambda, 2003).

Between 1942 and 1948, C. C. Li moved with the war but stuck to his profession as both a teacher and a researcher: From the Agricultural College of National Guangxi University (Shatang, near Chenzhou) in 1942 (Chen & Tai, 1998), to the National University of Nanking in Chengdu and then in Nanjing in 1943, finally to National Peking University in 1946 (Guo, 2016).

C. C. Li’s first book, *An Introduction to Population Genetics*, published in 1948 by the University Press, is a full summary of his teaching and research from that Experimental Farm on the campus in Beijing.

The period between 1942 and the end of 1948 is the first and only period of his direct contributions to genetics in China (Li, 1948; Gao, 2005; Chakravarti, 2004).

(2) 1949—The second choice: To stay in his motherland.

The second choice C. C. Li made was to stay in mainland China again with his people in the hope for the future of genetics in his motherland.

Even before Beijing announced its liberation on January 31st, 1949, there would be many reasons for him to join his wife’s family in the US. His wide communication with his former classmates, friends and colleagues, might have advised him to leave. It was also true that his wife made an effort to visit Shanghai in order to resume her US citizenship as early as in 1945 (Spiess, 1983). In addition Jerome, his younger brother, was at the time already at Oregon State University, US.

Three events, not firmly documented yet, could help explain why he chose to stay in mainland China:

First, it was he who invited a communist officer to the campus to give introduction to the “Liberation Region” around the time the liberation was announced (Ye, 1997).

Second, it was also true that “C. C. Li expressed a passionate wish to keep contributing to the new China’s science and education to Mr. Jianying Ye, then Mayor of Beijing” in January 1949 (Guo, 2016).

Third, his motivation to translate Mr. Trofim Lysenko’s representative book, *Heredity and Its Variability*, could be interpreted as a desire to know how genetics would develop in China (Guo, 2016).

It is absolutely sure: He embraced the best hope for a new China, as well as his dream to contribute to genetics in China.

(3) 1951—The third choice: To “escape” his motherland.

The third choice which had made a dramatic change in his life, as well as the most difficult choice he had ever made, led to C. C. Li’s “escaping” Beijing on March 8th, 1950.

It is not difficult to find out what was in his mind from his angry remarks: “Taking a purely academic issue as a political ‘hat’ for an enemy cannot be tolerated.” “Even with great patience, it is impossible for my colleagues and me to save genetics from extinction in China. In this case, one must declare that he is loyal to Lysenkoism, otherwise the only way is to escape”. He felt that there was no way to serve his motherland (Guo, 2008).

The situation for him—for most, if not all, of geneticists and genetics in the whole of China—rapidly deteriorated. Perhaps just through the process of translation of Lysenco’s book, C. C. Li realized that this “-ism” would totally ruin genetics in China, which totally disappointed him.

However, he did not give up. He refused to abandon his principles. He stood up as a MAN against the “Lysenko’s tide” which was flooding in the whole field of genetics. In the first half of 1949, C. C. Li publicly criticized the official propagandas on Lysenkoism as “totally wrong” and “completely unscientific” several times at meetings with teachers and students at the Department of Agriculture (Ye, 1997).

He might have become the first target, or at least one of the first *persona non grata*, of the campaign against the “counterrevolutionary genetics” (Wohleber, 1991). Lysenko’s followers, of course, further criticized “genetics”, “field design” and “biostatistics” taught by C. C. Li as “counterrevolutionary”, “bourgeois” “pseudo-science”, leading to the official banning of all the courses he had been teaching. His book, “Introduction to Population Genetics” was immediately “criticized” by an official journal.

As C. C. Li put it, “I was not allowed to teach. I was not allowed to do anything.” “No one was allowed to talk to me.” “I was persecuted.” (Wohleber, 1991; Srikameswaran, 2003).

Finally, C. C. Li resigned from his positions in the Department of Agronomy and the Agro-experimental Farm (Guo, 2008).

Really, we still don’t know what C. C. Li did was an “action” or a “reaction”, political or non-political. On one hand, it reminded him of the horrific events in the 1930s from a neighbouring country, and the fate-of-execution of many world-renowed geneticists there. As he put it: “Supporters of Lysenko genetics were called ‘patriots’, whereas Mendelian geneticists regarded as foreign spies, subject to the death penalty” (ASHG, on the cover, 2012).

On the other hand, as a man, he had to live. He had lost his job, he had lost his profession. He was unable to “teach” something he did not know, or did not want to know; he understood very well that Lysenkoism was something to “mislead the younger generations”. It was just an unacceptable fact by him as a teacher, let alone a researcher. The so-called “Li’s Defending Case” might have been very simple or non-political at all. It is just as simple and reasonable as a general “Dutch Goodbye” with the sole purpose not to bother or to involve anybody else.

However, objectively speaking, C. C. Li’s departure did disturb the leaders of the highest level who took immediate measures to suspend this situation. Perhaps it is fair to say, even if it cannot be said, that “Li’s Defending Case” had “saved” genetics in China and many Chinese geneticists, actually protecting—or at least “postponing”—many of them from the catastrophe at that time.

(4) After 1951: The fourth choice? No choice.

It would be “no choice”, if C. C. Li had made the fourth choice: To dedicate all his life to genetics. However, what else had been in his mind for the second half of his life?

In almost half of a century, he considered himself a “‘D.P.’—a displaced person” (Hart, 2002). He published 131 research and review papers in 47 years (from 1953 to 2000), with 25 of them published after his retirement. He delivered numerous speeches which received critical acclaim, such as “A Tale of Two Thermos Bottles: Properties of a Genetic Model for Human Intelligence”.

His 10 books (including 2 translated books), especially many textbooks, beginning with *Introduction to Population Genetics* published in Beijing in 1948, have become classics and been translated into several European and Asian languages and continued to inspire confidence and admiration of the students of generations all over the world to go into genetics.

More importantly, he has students all over the world, including 12 Ph.D. students whose relationship with C. C. Li was even closer than that between the “traditional Teachers and Students in China” (paper by three of his Ph.D, students).

It is well known that arriving in Pittsburgh, C. C. Li “almost singlehandedly started genetics research at Pitt”. He established one of the nation’s largest and longest-running programs for the training of genetic counselors—professionals who give information and advice to individuals with hereditary diseases—in the 1970s (Wohleber C, 1991), which serves to remind us how important and urgent it is for this moment for China to establish such a program.

To summarize his contribution to global genetics: He became the first Chinese American to be elected President of American Society of Human Genetics (ASHG), one of the most Influential organizations in genetics in the world; he delivered the well-known inaugural speech, entitled “The Diminishing Jaw of Civilized People”. He received the lifetime “Award for Excellence in Education”, the top honor by ASHG in 1998, which currently has been only awarded to 4 geneticists.

The most important issue is that he proved himself: Never a political “defender” to his motherland!

Many Chinese scholars and students from mainland, including the authors of this article, had been together with him many times since the 1980s. C. C. Li attended almost every one of the routine working dinner hosted by the American Society of Geneticists in the US during the annual meeting of the ASHG. He generally did not talk about his own experiences, nor the past, but he always asked about and gave his own frank comments on what was happening in his motherland, demonstrating his passions and concerns for genetics in China. It is not difficult for many of us to testify that even his “complaints” were no more “serious” than those of our own.

In the sense of science, we also have to admit that, most of the time, he was correct, even when expressed in his unique frankness and sharpness; for example, his sharp criticism on Lysenkoism and “eugenics”. How many of us would be able to confess that at least some of us did go “from the extreme left” (Lysenkoism to give up Mendelism) in the 1950s to “the extreme right” (eugenics to abuse Mendelism) in the 1980s as he criticized?

He did not just simply criticize us; he helped us. Let’s again take it as an example: No matter how he commented on it, he did help us with the translation of the Chinese version of the drafted Provisional Regulations on the Genetic Materials into English very carefully with many valuable and cordial suggestions. Now it could be said that he made great contribution to the English version of the Regulations (Chen and Tai, 1998).

It is also true that he did complain of the “official identification” of his “escaping” in “Li’s Defending Case”.

In a letter to Mr. Duzhuang Ye in August 1996, he wrote: “I don’t know if this conclusion is included in my personal documents”; “I personally feel that I should be rehabilitated. Even if this year I’m not rehabilitated, next year I’m not rehabilitated, in the end I should be rehabilitated; this should be inevitable”. He also said: “The past is past, the greatest pain in life is ‘separation and death’” (Guo, 2008).

In March 1997, he wrote another letter, as long as 17 pages, recollecting past events to Mr. Duzhuang Ye: “I wrote this letter for two days and fell into tears twice.” “There is a saying that the hero is not tearful, especially for an old man who has been through the vicissitudes of life for 85 years. Written for two days, fell two tears—its grievances, its pain, its sadness, its resentment was really touching (Ye, 1997).

May his soul rest in peace! He will live in our hearts forever.

## Electronic supplementary material

Below is the link to the electronic supplementary material.

Electronic supplementary material 1 (PDF 410 kb)
