# Supplementary material for: In memory of Prof. C. C. Li
Source: Protein Cell. 2018 Oct 23;10(6):389–92. doi: 10.1007/s13238-018-0583-y (PMC6538568; doi:10.1007/s13238-018-0583-y)
Supplement: Supplementary file 1 — Electronic supplementary material 1 (PDF 410 kb) [file 13238_2018_583_MOESM1_ESM.pdf]

Chronicle Vitae of Dr. Ching Chun Li (CC Li)

1912 On Oct. 27th, born in Taku, Tianjin

1925 Beginning his school education in Tientsin Anglo-Chinese College

1932 Entering the Agricultural College, University of Nanking, majoring in agronomy and forestry

Elected as President of the Student Union during the period

1936 Graduated with a Bachelor degree

1937 Entering Cornell University, USA, majoring in plant breeding and genetics

1940 Graduated with a Ph.D. degree

1940-1941 Postdoctoral trainings at the University of Chicago, Columbia University, North Carolina State College in the fields of mathematics, statistics, covariance analysis, probability theory, and Experimental Statistics

1941 Married with Ms. Clara A. Lem on Sept. 20th

The honeymoon trip taking 51 days on ocean because of the Japanese invasion

Arriving in Kowloon, Hong Kong on Dec. 6, trapped by nearly two months in Hongkong because of the "Pearl Harbor Attack" on Dec. 8

1942 Walking to Guilin in the war time for 38 days on the way in Jan and Feb

Jeff, their first son, born in Guilin in June

Teaching genetics, cytology and calculus at the Agricultural College of National Guangxi University (Shatang Town in the outskirts of Liuzhou, Guangxi)

Meeting Drs. Tao-Chiuh Hsu (TC Hsu) and Zudong Liu, China-born

geneticists.

1943 Jeff died in his arms on the way from Guilin to Chengdu in Summer

Invited to be Professor on Genetics and Biometry, University of Nanking

1945 Mrs. Li going to the US Consulate in Shanghai to resume her US citizenship
Carol, their daughter, born in Shanghai

1946 Taking the position of Professor of Agricultural College, National Peking University

Beginning to write textbooks as soon as he arrived in Beijing based on the teaching experiences and materials in Guangxi and Chengdu during the war

Serving as Director of the Department of Agronomy, and Director of its Agro-experimental Farm

1948 The first and most popular textbook, *Introduction to Population Genetics*, published by the National Peking University Press in Dec

The book immediately “criticized” by *The Rural News*, a journal by the Agricultural College of North China Union University

1949 Expressing his strong desire to serve his motherland (reasons to stay for the liberation) to General Jianying Ye, then Mayor of Beijing in Jan

All his courses, including Genetics, Field Design, and Biostatistics, officially banned in Sept

Concentrating on the translation of *Heredity and its Variability* by Lysenk TD

In Sept., “Resigning” from positions of Director of the Department of Agronomy, also Director of the Agro-experimental Farm

1950 In Feb, beginning the plan of “going away” (“Li Defending Case”) in mind, without telling his wife or anybody else

On March 10, leaving Beijing quietly, taking two days’ train to Shanghai

On March 12, arriving in Hong Kong immediately after crossing the Luohu Bridge

Soon after arriving, sending a letter to Prof. Theodosius Dobzhansky, a world-renowned geneticist in the US, later published in *The Journal of Heredity* entitled *Genetics Dies in China*, thus “Li Case” revealed to the outside world
(This might lead to a series of assistant efforts by Prof. H. Muller, a winner of the 1946 Nobel Prize in Physiology or Medicine, as well as the invitation from Taipei)

In March, visited by a “professor of Tsinghua University”, or an anonymous VIP, from Beijing, exchanging information and his situation/motivation frankly for the whole afternoon that day
(This might be linked to the first official report of the “Li Case” by Mr. Junrui Qian, then Deputy Minister of Education, to the highest lever leaders in Beijing in April)

In April, officially identified as a “counter-revolutionary defending event” by the authorities of Agricultural College, Peking University

In April, refusing an offer of professorship by President of the National Taiwan University

In May, Mr. Qiaomu Hu, then vice Minister of the Propaganda, paying attention to “Li Defending Case” in May

in June, Mr. Ji Ma, then an Assistant Professor of Department of Agriculture and Forestry, Beijing Agricultural University, writing the first true story about the “Li Defending Case”, submitted to then Premier Zhou Enlai and then Chairman Mao Tze Tung. Both giving comments on the Case respectively

1951 On April. 7, Prof. H. Muller arriving in Hong Kong, while Nature publishing Prof. K. Mather’s review on Li’s first textbook, entitled the *Chinese textbook on population genetics*

On April 30, with the help of Mr. Muller, a “US Special Visa” finally issued to him

On May 5, arriving in Portland, Oregon, USA

On May 21, Li’s Family arriving in Pittsburgh, and meeting Dr. Newton Morton, then

a Ph.D. student in genetics

Beginning to serve as a Research Fellow on Biostatistics, University of Pittsburgh

In Sept. Steve Muller Li, their second son, born

1953 Chinese version of the book, *Soviet genetics and World Science* (by Julian Huxley), published in Taipei by the Chinese Culture Association Publishing

1954 Beginning to serve as Assistant Professor at Graduate School of Public Health, University of Pittsburgh

The famous "ITO Method" was proposed in the article, *The derivation of joint distribution and correlation between relatives by the use of stochastic matrice*s, published in *The Journal of Biostatistics*

1955 The fourth book, *Population Genetics*, reprinted by the University of Chicago Press

Introduced by Muller and invited by Director of CSHL to participate in Cold Spring Harbor seminar, "Population Genetics: The nature and cause of genetic variants in population"

Mid-1950s Two widely applied principles for clinical trials proposed: Randomized Double-Blind Placebo-controlled Trial

1958 Beginning to serve as Associate Professor, University of Pittsburgh

1959 The fifth book, *Numbers from Experiments*, published by Boxwood Press

1960 Beginning to serve as Professor of Biostatistics, University of Pittsburgh on April 8

Elected as President of the American Society of Human Genetics, one of the most Influential organizations in genetics in the world, delivering the well-known inaugural speech, entitled "The Diminishing Jaw of Civilized People"

In Dec., invited by the American Association for the Advancement of Science (AAAS) to assess the science of the mainland China

Eight papers, such as *Simplified method estimation the MNS gene frequencies* in the journal of *Ann Hum Genet*

1961 The sixth book, *Human Genetics: Principles and Methods*, published by McGraw-Hill Book Co. Later translated into Italian, Spanish, and other languages

1962 Elected as a member of Academia Sinica in Taipei

1964 The seventh book, *Introduction to Experimental Statistics,* published by McGraw-Hill Book Co

1965 In Aug., invited to the Mendel Memorial Conference in Brno, Czech Republic

1967 One of the important articles, *Genetic Equilibrium under Selection* published

1968 In Summer, invited to the discussion about genetic and anthropological studies on Olympic Athletes

1969 Beginning to serve as Director of Department of Biostatistics and Human Genetics, University of Pittsburgh

1970 Named "Statistician of The Year" by the American Statistical Association

The famous "story" entitled *A Tale of Two Thermos Bottles: Properties of a Genetic Model for Human Intelligence*

Another well-known article, *Intelligence: Genetic and Environmental Issues* published

1974 In March, invited to the Second National Congress of the Mexican Genetic Society

1975 The eighth book, *First Course in Population Genetics*, published by Boxwood

The ninth book, *Path Analysis* --- *a Primer*, published

Beginning to serve as Professor of Statistical Biology and Human Genetics, Department of Human Genetics, Graduate School of Public Health, University of

Pittsburgh

1976 Serving as an expert of the US Congress and Commission for the Control of Huntington’s Disease

1982 The tenth book, *Analysis of unbalanced data:A Pre-Program Introduction* published by Cambridge University Press

Beginning to serve as Emeritus Professor of Statistical Biology and Human genetics, Department of Human Genetics, Graduate School of Public Health, University of Pittsburgh

On Oct. 3 – 5, “The Medal of Pittsburgh University” awarded at The 70th Anniversary of the Birth of C.C. LI and Conference on Human Population Genetics

1983 On Jan. 1, formally retired at the age of 70

1983-2002 A total of 25 papers published after retiring

1987 Publishing an article in memory of the 100th Anniversary of Vavilov Nikolay Ivanovich, a well-known geneticist in Russian who was forced to die

1995 A Chinese Version of the book, *An Introduction to Experimental Genetics* (1964 Edition), published by Heilongjiang Education Press

1996 An article entitled *Population genetics of coincidental DNA match* published in *The Journal of Human Biology*, named “The Best Paper of the Year” and awarded “The Gabriel W. Lasker Award”

1998 A “Lifetime Distinguished Education Award” awarded by the American Society of Human Genetics

2001 The book, *First Course in Population Genetics*, presented to China Agricultural University as a gift

2002 $1 million donated by the Li’s to establish “The Human Genetics Research and Education Support Fund”

2003 On Oct. 20, passing away in the residence in Pittsburgh, at the age of 91

PUBLICATION LIST OF PROFESSOR CHING CHUN LI

1.**Li CC**. A direct proof of the relation between genotypic mating correlation and gametic uniting correlation in equilibrium populations. *J Hered* 1953; 44:3940.

2.**Li CC**. On an equation specifying equilibrium populations. *Science* 1953; 117:378-379.

3.**Li CC**. Is RH facing a crossroad? A critique of the compensation effect. *Am J Natural* 1953; 87:257-261.

4.**Li CC**. Some general properties of recessive inheritance. *Am J Hum Genet* 1953; 5:269-279.

5.**Li CC**, Horvitz DG. Some methods of estimating the inbreeding coefficient. *Am J Hum Genet* 1953; 5:107-117.

6.Glass HB, **Li CC**. The dynamics of racial intermixture - an analysis based on the American Negro. *Am J Hum Genet* 1953; 5:1-20.

7.**Li CC**, Sacks L. The derivation of joint distribution and correlation between relatives by the use of stochastic matrices. *Biometrics* 1954; 10:347-360.

8.**Li CC**. The correlation between parents and offspring in a random mating population. *Am J Hum Genet* 1954; 6:383-386.

9.**Li CC**. The stability of an equilibrium and the average fitness of a population. *Am J Natural* 1955; 89:281-296.

10.**Li CC**. A diagrammatic representation of the sum of squares and products. *J Am Stat Assoc* 1955; 50:1056-1063.

11.**Li CC**. The concept of path coefficient and its impact on population genetics. *Biometrics* 1956; 12:190-210.

12.**Li CC**. The components of sampling variance of ABO gene frequency estimates. *Am J Hum Genet* 1956; 8:133-137.

13.**Li CC**. Repeated linear regression and variance components of a population with binomial frequencies. *Biometrics* 1957; 13:225-234.

14.**Li CC**. The genetic variance of autotetraploids with two alleles. *Genetics* 1957; 42:583-592.

15.Lewis JH, **Li CC**. Genetic considerations in hemophilia A and B. *Proceedings of X* International Congress of Genetics 1958; 2:168.

16.**Li CC**. A clinical study of the comparative effect of nitrogen mustard and DON in patients with bronchogenic carcinoma, Hodgkin's Disease, lymphosarcoma, and melanoma. *J Natl Cancer Inst* 1959; 22:433-439.

17.**Li CC**. Notes on relative fitness of genotypes that form a geometric progression. *Evolution* 1959; 13; 564-567.

18.DeGroot MH, **Li CC**. Simplified method estimation the MNS gene frequencies. *Ann Hum Genet* 1960; 24:109-115.

19.**Li CC**. A diagrammatic representation of the proportions of genotypes and phenotypes in a panmictic population. *Ann Hum Genet* 1960; 24:117-119.

20.**Li CC**. McGraw-Hill Encyclopedia of Science and Technology. *Biometrics* 1960; 1:223-232.

21.**Li CC**. McGraw-Hill Encyclopedia of Science and Technology. *Hum Genet* 1960; 2:497-513.

22.**Li CC**. McGraw-Hill Encyclopedia of Science and Technology. *Population Genet* 1960; 2:506- 512.

23.**Li CC**. The diminishing jaw of civilized people. *Am J Hum Genet* 1961; 13:1-8.

24.**Li CC**. Reinhold Encyclopedia of Biological Sciences. *Population Genet* 1961; 818-822.

25.**Li CC**. Genetical methods for epidemiological investigations: A synthesis. *Ann NY Acad Sci* 1961; 91:806-812.

26.**Li CC**. Genetics (in Communist China). *The China Quarterly*. London. 1961; 6:144-152.

27.Smith CAB, **Li CC**. Estimation of linkage using Iweedie’s method. Second International Conference on Human Genetics (Rome), *Proceedings*. 1961; 175-177 (E146-147).

28.Wald N, Borges WH, **Li CC**, Turner JH, Harnois MC. Leukemia associated with mongolism. *Lancet* 1961; i:1228.

29.**Li CC**. Estimating the degree of mating propensity. *Evolution* 1961; 15:543-544.

30.**Li CC**. On "reflexive selection". *Science* 1962; 136:1055-1056.

31.**Li CC**. Blood groups and disease. *Lancet* 1962; 2:98-99.

32.**Li CC**. Decrease of population fitness upon inbreeding. *Proc Natl Acad Sci USA* 1963; 49:439- 445.

33.**Li CC**. Genetic aspects of consanguinity. *Am J Med* 1963; 34:702-714.

34.**Li CC**. The way the load ratio works. *Am J Hum Genet* 1963; 15:316-321.

35.**Li CC**. Equilibrium under differential selection in the sexes. *Evolution* 1963; 17:493-496.

36.**Li CC**, Lewis JH, Didisheim P, Ferguson IH. Genetic considerations in familial hemorrhagic disease. I. The sex-linked recessive disorders, hemophilia and RC deficiency. *Am J Hum Genet* 1963; 15:5341.

37.Boyd WC, **Li CC**. Rates of selective action on unifactorial and multifactorial traits. *Am J Physic Anthrop* 1963; 21:521-526.

38.Kaplan B, **Li CC**, Wald N, Borges WH. ABO frequencies in mongols. *Ann Hum Genet* 1964; 27:405-412.

39.**Li CC**. Estimate of recessive proportion by first appearance time. *Ann Hum Genet* 1964; 28:177-180.

40.**Li CC**. Two additional views of linear regression coefficients. *Am Stat* 1964; 18:27-28.

41.**Li CC**. The hemophilia gene in the population. *Proceedings of International Conference on. Hemophilia.* University of North Carolina Press, Chapel Hill. 1964.

42.**Li CC**. Segregation of Ellis-van Creveld syndrome as analyzed by the first appearance method. *Am J Hum Genet* 1965; 17:343-351.

43.O'Brien WM, **Li CC**, Taylor FH. Penetrance and the distribution of sib-pair types, exemplified by taste ability and rheumatoid arthritis. *J Chron Dis* 1965; 18:675-680.

44.**Li CC**. Mutation as a dynamic agent in Mendelism. *Mutation in Populations*. Prague 1966; 13.

45.**Li CC**. A new method of studying Mendelian segregation in man. *Mutation in Populations*. Prague 1966; 155-166.

46.DeGroot MH, **Li CC**. Correlation between similar sets of measurements. *Biometrics* 1966; 22:781-790.

47.**Li CC**. Castle's early work on selection and equilibrium. *Am J Hum Genet* 1967; 19:70-74.

48.**Li CC**. Fundamental theorem of natural selection. *Nature* 1967; 214:505-506.

49.**Li CC**. The maximization of average fitness by natural selection for a sex-linked locus. *Proc Natl Acad Sci USA* 1967; 57:1260-1261.

50.**Li CC**. Genetic equilibrium under selection. *Biometrics* 1967; 23:397-484.

51.**Li CC**, Mantel N. A simple method of estimation the segregation ratio under complete ascertainment. *Am J Hum Genet* 1968; 20:61-81.

52.**Li CC**. Fisher, Wright, and path coefficients. *Biometrics* 1968; 24:471483.

53.Rao BR, Garg M, **Li CC**. Correlation between the sample variances in a singly truncated bivariate normal distribution. *Biometrics* 1968; 55:433-436.

54.**Li CC**. Increment of average fitness for multiple alleles. *Proc Natl Acad Sci USA* 1969; 62:395- 398.

55.**Li CC**. Population subdivision with respect to multiple alleles. *Ann Hum Genet* 1969; 33:23-29.

56.Falk C, **Li CC**. Negative assortative mating: exact solution to a simple model. *Genetics* 1969; 62:215-223.

57.**Li CC**, Falk C. Random mating between two sets of genotypes. *Biometrische Zeitschrift*, 1969; 11:361-373.

58.Li CC. Table of variance of ABO gene frequency estimates. *Ann Hum Genet* 1970; 34:189-194.

59.**Li CC**. Unsymmetric equilibria under two-locus symmetric selection model. *J Hered* 1971; 62:47-48

60.Waller JH, Rao BR, **Li CC**. Heterogeneity of childless families. *Soc Biol* 1973; 20:133-138.

61.Rao BR, Mazumdar S, Waller JH, **Li CC**. Correlation between the numbers of two types of children in a family. *Biometrics* 1973; 29:271-279.

62.Mantel N, **Li CC**. Estimation and testing of a measure of non-random mating. *Ann Hum Genet* 1974; 37:445-454.

63.**Li CC**. Assortative mating in man. *Proc Second Mexican Society of Genetics*. 1974; 48-108.

64.**Li CC**. De quelques proprietes generales de la genetique quantitative appliquee aux populations humaines. *L'Orthodontie Francaise, Extraits du.* 1975; 46:55-69.

65.**Li CC**. Path coefficients and derivatives. *Biometrische Zeitschrift* 1975; 17:213-215.

66.**Li CC**, Mazumdar S, Rao BR. Partial correlation in terms of path coefficients. *Am Statist* 1975; 29:89-90.

67.**Li CC**, Mazumdar S. Analysis of dichotomized factorial data. *J Chron Dis* 1976; 29:355-370.

68.**Li CC**. The testing of dominants for heterozygosity. *Ann Hum Genet* 1976; 40:183-190.

69.**Li CC**, Mazumdar S. Analysis of dichotomized factorial data. *J Chron Dis* 1976; 29:355-370.

70.**Li CC**. Separation of common environment and dominance effects with classic kinship correlation models. *Soc Biol* 1977; 4:259-266.

71.**Li CC**. On measuring genetic distance by selection intensity. *Ann Hum Genet* 1978; 41:501- 504.

72.**Li CC**. Progress of the kinship correlation models. In Morton NE Chung CS, eds: *Genetic Epidemiology*. Academic Press, New York, 1978, 55-86.

73.Li CC. The genetical and environmental contributions to kinship correlations. *Proceedings of the Golden Jubilee of Academia Sinica*, Mielke JM, Crawford MH, eds: Taipei Taiwan. 1978; 397-417.

74.**Li CC**. Half sib analysis for quantitative data. In J.M. Mielke, M.H. Crawford, eds: *Current Developments in Anthropological Genetics*. Plenum Publishing Corp., New York, 1978; 1:373-396.

75.Mazumdar S, **Li CC**, Bryce R. Correspondence between a linear restriction and a generalized inverse in linear model analysis. *Am Statist* 1980: 34:103-105.

76.**Li CC**, Mazumdar S. A type of orthogonal contrasts for unbalanced data. *Biometrics* 1981; 23:645-651.

77.Rao BR, **Li CC**. The geometry of path coefficients and correlations. *Biometrical J* 1982; 24(8):673-378.

78.Chakravarti A, **Li CC**. The effect of linkage on paternity calculations. In: *Inclusion Probabilities in Parentage Testing*. Am Assoc Blood Banks, Arlington, VA., 1983; 411-422.

79.**Li CC**, Chakravarti A. On the exclusion and paternity probabilities. In: *Inclusion Probabilities in Parentaze Testing*. Am Assoc Blood Banks. Arlington, VA. 1983; 609-622.

80.Chakravarti A, **Li CC**. The probability of exclusion based on the HLA locus. *Am J Hum Genet* 1983; 35:1048-1052.

81.Chakravarti A, **Li CC**, Buetow KH. Estimation of the marker gene frequency and linkage disequilibrium from conditional marker data. *Am J Hum Genet* 1984; 36:177-186.

82.Chakravarti A, **Li CC**. Estimating the prior probability of paternity from the results of exclusion tests. *Forensic Sci Internatl* 1984; 24:143-147.

83.**Li CC**. Some methodological developments in genetic epidemiology. In DC Rao et al, eds : *Genetic Epidemiology of Coronary Heart Disease; Past, Present and Future*. Alan R. Liss, New York, 1984; 159-172.

84.**Li CC**. Evaluation of direct and joint effects in a causal system. *Proceedings of Second Symposium on Mathematical Statistics and Probability*. Taipei, Taiwan. 1984.

85.**Li CC**, Chakravarti A. Basic fallacies in the formulation of the paternity index. *Am J Hum Genet* 1985; 37:809-818.

86.**Li CC**, Chakravarti A. Some fallacious thinking about the paternity index: A reply to Dr. Jask Valentin's comments. *Am J Hum Genet* 1986; 38:586-589.

87.**Li CC**. Inbreeding and the balance between selection and mutation. In Gershowitz H, Rucknagel DL Tashian RE, eds: *Evolutionary Perspectives and the New Genetics*. Alan R. Liss, New York, 1986; 31-44.

88.**Li CC**. A method of subdividing genetic data into self-contained subsets. *Ann Hum Genet* 1986; 50:259-270.

89.**Li CC**. The effect of father's education on child's cognitive ability. *Soc Biol* 1986; 33:316-321.

90.**Li CC**. A genetical model for emergencies. In memory of Laurence H. Snyder (1901-1986). *Am J Hum Genet* 1987; 41:517-523.

91.**Li CC**. Lysenkoism in China. *J Hered* 1987; 78:339-340.

92.**Li CC**. To the memory of the fallen: Nikolai Invanovich Vavilov (1887-1943). *J Hered* 1987; 78:343.

93.**Li CC**, Chakravarti A, Halloran SL. Estimation of segregation and ascertainment probabilities by discarding the single probands. *Genet Epidemiol* 1987; 4:185-191.

94.Chakravarti A, Badner JA, **Li CC**. Tests of linkage and heterogeneity in Mendelian diseases using identity by descent scores. *Genet Epidemiol* 1987; 4:255-266.

95.**Li CC**. Steinberg's new paternity probability. *Am J Hum Genet* 1988; 42:390-391.

96.**Li CC**. Pseudo random mating populations: In celebration of the 80th anniversary of the Hardy- Weinberg Law. *Genetics* 1988; 119:731-737.

97.**Li CC**, Chakravarti A. An expository review of two methods of calculating the paternity probability. *Am J Hum Genet* 1988; 43:197-205.

98.Majumder PP, Das SK, **Li CC**. A genetical model for vitiligo. *Am J Hum Genet* 1988; 43:119-125.

99.**Li CC**. Method of path coefficients: A trademark of Sewall Wright. *Hum Biol* 1991; 63:1-17.

100.**Li CC**. Genetics of subdivided populations and its relationships with certain measures of association. *Genet Epidemiol* 1991; 8:1-11.

101.**Li CC**, Weeks DE, Chakravarti A. A similarity of DNA fingerprints due to chance and relatedness. *Hum Hered* 1993; 43:45-52.

102.**Li CC**, Chakravarti A. DNA profile similarity in a subdivided population. *Hum Hered* 1994; 44:100-109.

103.Weeks DE, Young A, **Li CC**. DNA profile match probabilities in a subdivided population: When can subdivision be ignored? *Proc Natl Acad Sci USA* 1995; 92:12031-12035.

104.**Li CC**. Population genetics of coincidental DNA matches. *Hum Biol* 1996; 68:167-184.

105.Guo SW, Zheng CJ, **Li CC**. “Gene war of the century”? *Science* 1997; 278:1693-1694.

106.Guo SW, Zheng CJ, **Li CC**. Dilemma over genetics and population in China. *Nature* 1998; 394:313-314.

107.**Li CC**. 1998 ASHG Award for Excellence in Education. Remarks on receiving the ASHG award: science and science education. *Am J Hum Genet* 1999; 64:16-17.

108.**Li CC**. Progressing from eugenics to human genetics. Celebrating the 70th birthday of professor Newton E. Morton. *Hum Hered* 2000; 50:22-33.

Reviews

1.**Li CC**. Genetics of population structure. *J Hered* 1955; 46:57-58.

2.**Li CC**. Human heredity. *J Hered* 1955; 46:283-284.

3.**Li CC**. Genetic variability in populations. *Quart Rev Biol* 1957; 32:167-170.

4.**Li CC**. An introduction to genetic statistics. *Am J Hum Genet* 1958; 10:72-75.

5.**Li CC**. The genetic basis of selection. *Am J Hum Genet* 1959; 11:84-86.

6.**Li CC**. The analysis of variance. *Quart Rev Biol* 1961; 2:154.

7.**Li CC**. Modern probability theory and its applications. *Quart Rev Biol* 1961; 3:245-246.

8.**Li CC**. Genetic mechanisms in human disease; Chromosomal aberrations. *J Am Med Assoc* 1962; 181:455.

9.**Li CC**. Genetical variation in human populations. *JAMA* 1962; 181:567.

10.**Li CC**. Statistical processes of evolutionary theory. *Am J Hum Genet* 1962; 14:438-439.

11.**Li CC**. Methodology in human genetics. *Science* 1962; 138:807-808.

12.**Li CC**. Changing perspectives on the genetic effects of radiation. *JAMA* 1963; 185:675.

13.**Li CC**. Elementary medical statistics. *JAMA* 1963; 186:1027.

14.**Li CC**. Elements of medical statistics. *JAMA* 1964; 188:242-243.

15.**Li CC**. The effects of inbreeding on Japanese children. *JAMA* 1966; 195:974.

16.**Li CC**. Human genetics and public health. *Eugenics Quart* 1966; 13:169-170.

17.**Li CC**. Research in population genetics of primitive groups. *Eugenics Quart* 1966; 13:170-171.

18.**Li CC**. The correlation between relatives on the supposition of Mendelian inheritance. *Quart Rev Biol* 1967; 42:425-426.

19.**Li CC**. A programmed introduction to statistics. *Technometrics* 1968; 10:411.

20.**Li CC**. The groundwork for a genetic study. *Science* 1969; p. 163.

21.**Li CC**. Topics in population genetics. *Am J Hum Genet* 1969; 21:410-411.

22.**Li CC**. "Simplified" population genetics. *J Hered* 1969; 60 :238.

23.**Li CC**. More fuel for the genetic-load stove. *J Hered* 1970; 61:105-106.

Books

1.*Introduction to Population Genetics.* National Peking University Press. 1948.

2.*Heredity and its Variability* (by Lysenko TD). Chinese translation, New China Book Co. 1949.

3.*Soviet Genetics and World Science* (by Julian Huxley). Chinese translation, Taipei, Taiwan. 1953

4.*Population Genetics.* University of Chicago Press. 1955.

5.*Numbers from Experiments*. Boxwood Press. 1959.

6.*Human Genetics, Principles and Methods*. McGraw-Hill Book Co. 1961.

7.*Introduction to Experimental Statistics*. McGraw-Hill Book Co. 1964.

8.*First Course in Population Genetics*. Boxwood Press. 1975.

9.*Path Analysis, A Primer.* Boxwood Press. 1975.

10.*Analysis of Unbalanced Data A Pre-Program Introduction*. Cambridge University Press. *Cambridge, England. 1982.*
